# Lack of Association of *ACE2* G8790A Gene Mutation with Essential Hypertension in the Chinese Population: A Meta-Analysis Involving 5260 Subjects

**DOI:** 10.3389/fphys.2012.00364

**Published:** 2012-09-12

**Authors:** Yan-yan Li

**Affiliations:** ^1^Department of Geriatrics, First Affiliated Hospital of Nanjing Medical UniversityNanjing, China

**Keywords:** angiotensin converting enzyme 2, gene polymorphism, G8790A, hypertension, meta-analysis

## Abstract

**Background:** The *angiotensin converting enzyme 2* (*ACE2*) G8790A gene polymorphism has been associated with the susceptibility to essential hypertension (EH), but the results are disputable. **Objective and Methods:** To investigate the relationship between the *ACE2* G8790A gene polymorphism and EH, eight separate studies with 5260 subjects were meta-analyzed. The pooled odds ratio (OR) and its corresponding 95% confidence interval (CI) were calculated by a random effect model. **Results:** In the *ACE2* G8790A gene polymorphism and EH meta-analysis in a Chinese population, no significant association was found between the *ACE2* G8790A gene polymorphism and EH (OR: 1.03, 95% CI: 0.87–1.21, *P* = 0.76). In the stratified analysis by gender, no significant risk was found among males (OR: 1.06, 95% CI: 0.82–1.36, *P* = 0.66) or females (OR: 0.98, 95% CI: 0.77–1.24, *P* = 0.85). Under a dominant model of inheritance in the female subgroup, the pooled OR for the GG/GA + AA value was 1.01 (95% CI: 0.82–1.25, *P* = 0.92). Under a recessive model of inheritance in the female subgroup, the pooled OR for the AA/AG + GG value was 0.93 (95% CI: 0.50–1.73, *P* = 0.83). **Conclusion:** The current meta-analysis suggested that the *ACE2* G8790A gene polymorphism might not be related to the increased EH risk in the Chinese population.

## Introduction

The gene polymorphism of *angiotensin converting enzyme 2* (*ACE2*) has been associated with the pathogenesis of essential hypertension (EH). *ACE2*, a homolog of *ACE* discovered by Donoghue et al. (2000) and Tipnis et al. (2000), has a catalytic domain 42% similar with that of *ACE*. *ACE2* is mainly expressed in the kidney, but is also found in the heart, testes, blood vessels, lungs, brain, nose, mouth, gastrointestinal tissues, and so on. In contrast to *ACE*, *ACE2* is not obviously expressed in any of these tissues, but is mostly expressed in endothelial cells. *ACE2* is located in the Xp22 chromosome with 18 exons. *ACE2* hydrolyzes angiotensin (Ang) I and generates Ang 1–9. Ang 1–9 is resolved into Ang 1–7, whose physiological activities are contrary to Ang II. Ang 1–7 can relax vessels, decrease the blood pressure, as well as inhibit the proliferation of cardiac myocytes, myocardium fibroblasts, and vascular smooth muscle cells. Hence, *ACE2* and *ACE* exhibit mutual antagonism. *ACE2* is supposed to demonstrate a protective function in the regulation of heart and blood pressures. Consequently, *ACE2* may become a new treatment target for cardiovascular diseases.

*Angiotensin converting enzyme 2 gene*, located in X chromosome, spans ∼2.4 kb and contains 18 exons. The G8790A polymorphism was at fourth base in the third intron adjacent to exon. The 8790th base guanine (G) was substituted by adenine (A) which resulted in the mRNA montage changes and *ACE2* gene expression.

The association of the *ACE2* G8790A gene polymorphism with EH significantly differed among various ethnicities. Benjafield et al. (2004) have found no association between the *ACE2* G8790A gene polymorphism and EH in Australian individuals by a polymerase chain reaction-restriction fragment length polymorphism analysis (Benjafield et al., 2004). Zhong et al. (2006) have found that females and individuals carrying only the G allele had significantly higher risks for increased diastolic blood pressure (DBP). The *ACE2* G8790A polymorphism is also concluded to be associated with hypertension in Han-Chinese patients with metabolic syndromes (Zhong et al., 2006). Jiang et al. (2010) found that the *ACE2* G8790A polymorphism might be associated with EH. They reported that male populations with G allele and female population with GG genotype had higher risk of EH in China (Jiang et al., 2010). However, Si et al. (2009) reported that the A allele increased the EH risk only in the male Chinese population. They found no significant association of *ACE2* G8790A gene polymorphism and EH in the female Chinese population. Zhou and Yang (2009) performed a meta-analysis on the *ACE2* G8790A polymorphism with Chinese Han EH and they concluded that *ACE2* G8790A polymorphism might not be genetic risk factor for EH in a Chinese Han population. In contrast, Lu et al. (2012) also conducted a similar meta-analysis and they found that *ACE2* G8790A polymorphism was probably a genetic risk factor for EH across different ethnic populations in female subjects and in Han-Chinese male subjects.

The present meta-analysis, which involved 5260 participants, was performed to obtain a more precise and comprehensive estimation of the association between the *ACE2* G8790A gene polymorphism and EH in the Chinese population.

## Materials and Methods

### Publication search and inclusion criteria

The electronic databases PubMed, Embase, Web of Science, China Biological Medicine Database, and China National Knowledge Infrastructure were searched in the current meta-analysis using the medical subject heading “hypertension,” “ACE2,” and “gene polymorphism.” The last research was performed on July 23, 2012. The range of publication years was from 2005 to 2010.

The inclusion criteria were as follow: (a) evaluation of the association between the *ACE2* G8790A gene polymorphism and EH; and (b) the EH diagnosis was in line with the 1999 EH diagnosis criteria of the World Health Organization as systolic blood pressure (SBP) ≥140 mmHg, DBP ≥90 mmHg. Secondary hypertension was not included in the present investigation.

### Data extraction

Data were collected according to a standard protocol. Repeated publications, those that violate any inclusion criterion, and/or those deviating from the Hardy–Weinberg equilibrium (HWE) were excluded. If different articles conveyed the same results, the results were considered only once in the current meta-analysis. The information extracted from literature included the name of the first author, year of publication, region, number of genotypes, total number of cases, and controls listed in Tables S1 and S2 in Supplementary Material. Given that *ACE2* is located in the X chromosome, the meta-analysis was classified into male and female subgroups.

### Statistical methods

The strength of association between the *ACE2* G8790A gene polymorphism and EH was assessed by the odds ratio (OR) corresponding to a 95% confidence interval (CI). The chi-square-based *Q*-test was used to check the heterogeneity assumption (significance set at *P* < 0.05; Cochran, 1968). Upon determination of heterogeneity among studies, the random-effects model was used to estimate the pooled OR by the DerSimonian and Laird method (DerSimonian and Laird, 1986). Otherwise, the pooled OR was estimated using the fixed-effects model (the Mantel–Haenszel method; Mantel and Haenszel, 1959). *I*^2^ was used to assess the heterogeneity. The lower was the *I*^2^, the smaller was the heterogeneity. Fisher’s exact test was used to assess the HWE (significance set at *P* < 0.05). The potential publication bias was estimated by the funnel plot. Egger et al.’s (1997) linear regression test of the natural logarithm scale of the OR (significance set at *P* < 0.05) was used to assess the funnel plot asymmetry. The STATA 10.0 software (StataCorp, College Station, TX, USA) was used to perform all statistical analyses.

## Results

### Study characteristics and meta-analysis results

**Figure 1:**
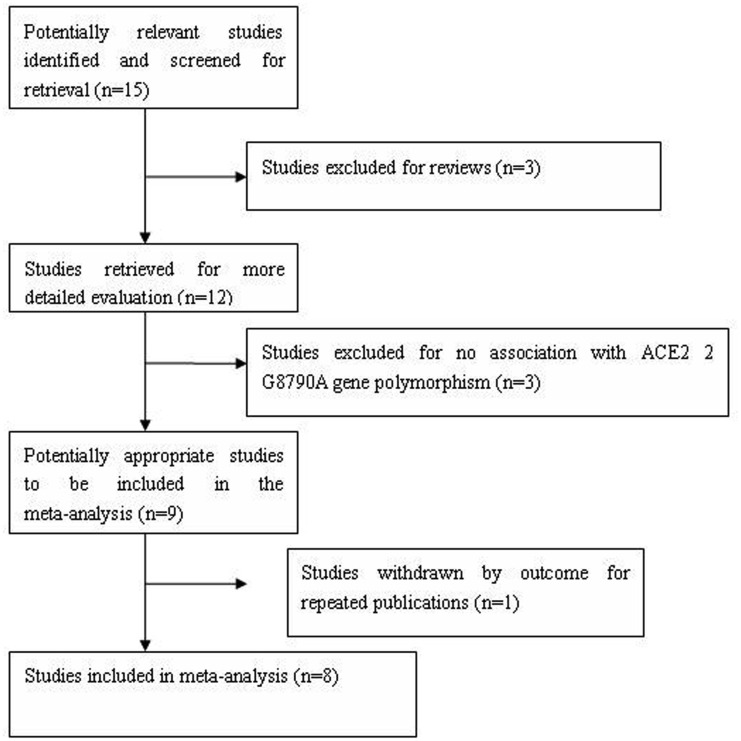
**Flow diagram of articles selection process for *ACE2* G8790A gene polymorphism and EH risk meta-analysis**.

A total of 15 papers were retrieved by the literature search, and eight of the papers fitted the study selection criteria. Of the seven excluded studies, three papers were reviews, three had nothing about the ACE 2 G8790A gene polymorphism, and one was a repeated publication (Zhang et al., 2008). In the female subgroup, four papers deviating from the HWE were excluded (Liu et al., 2005; Niu et al., 2007; Si et al., 2009; Zhang et al., 2010; Figure 1). In total, the data from the eight studies were gathered from 3090 men and 2170 women from three ethnicities. There were 2769 EH patients and 2491 controls. In the male subgroup, there were 1670 EH patients and 1420 controls. In the female subgroup, there were 1099 EH patients and 1071 controls. The OR values differed among the eight studies, and some of which approved that the A allele of G8790A increased the EH risk. However, the results of other studies were unclear and had to be integrated to reach a valuable conclusion. The three ethnicities included Han, Dongxiang and Li (Tables S1 and S2 in Supplementary Material; Liu et al., 2005; Hang et al., 2006; Yi et al., 2006; Fan et al., 2007; Niu et al., 2007; Si et al., 2009; Jiang et al., 2010; Zhang et al., 2010).

**Figure 2:**
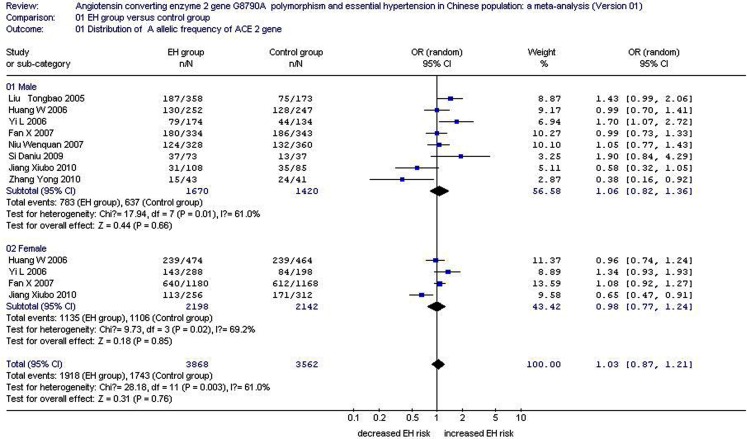
**Forest plot of EH associated with ACE2 G8790A gene polymorphism under an allelic genetic model stratified by gender (distribution of A allelic frequency of ACE2 gene)**.

In the present *ACE2* G8790A gene polymorphism and EH meta-analysis, under an allelic genetic model of inheritance, the distribution of the A allelic frequency was 0.496 for the EH group and 0.489 for the control. Figure 2 shows the summary OR of the distribution of the A allelic frequency, which was 1.03 (95% CI: 0.87–1.21) by the random effects model. The heterogeneity comparisons significantly differed (*P* = 0.003, *I*^2^ = 61.0%), but the EH and control groups did not (*P* = 0.76; Table 1; Figure 2).

**Table 1:** **Summary of meta-analysis of association of *ACE2* G8790A gene polymorphism and EH risk in the Chinese population**.

Gender	Pooled OR (95% CI)	*Z* (*P*)	*I*^2^ (%)
Allelic genetic model	1.03 (0.87–1.21)	0.31 (*P* = 0.76)	61.0
Male subgroup	1.06 (0.82–1.36)	0.44 (*P* = 0.66)	61.0
Female subgroup	0.98 (0.77–1.24)	0.18 (*P* = 0.85)	69.2
Dominant genetic model for female	1.01 (0.82–1.25)	0.10 (*P* = 0.92)	50.7
Recessive genetic model for female	0.93 (0.501–1.73)	0.22 (*P* = 0.83)	85.5

*CI, confidence interval; OR, odds ratio; Allelic genetic model: distribution of A allelic frequency of *ACE2* gene; Dominant genetic model: GG/GA + AA; Recessive genetic model: AA/GA + GG*.

The summary OR of the distribution frequency of the A allele in the male subgroup was 1.06 (95% CI: 0.82–1.36), as shown in Figure 2. In this subgroup, the distribution of the A allelic frequency was 0.469 for the EH group and 0.449 for the control. The heterogeneity among the eight studies significantly differed (*P* = 0.01, *I*^2^ = 61.0%), but the EH and control groups did not (*P* = 0.66; Table 1; Figure 2).

The summary OR of the distribution frequency of the A allele in the female subgroup was 0.98 (95% CI: 0.77–1.24), as shown in Figure 2. In this subgroup, the distribution of the A allelic frequency was 0.516 for the EH group and 0.516 for the control. The heterogeneity among the four studies also significantly differed (*P* = 0.02, *I*^2^ = 69.2%), but the EH and control groups did not (*P* = 0.85; Table 1; Figure 2).

**Figure 3:**
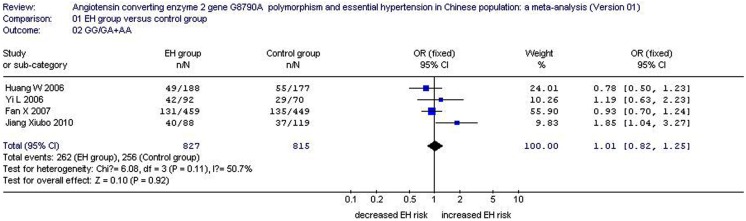
**Forest plot of the dominant genetic model of EH associated with *ACE2* G8790A gene polymorphism in female population (GG/GA + AA value of angiotensin converting enzyme 2 gene)**.

Under a dominant model of inheritance in the female subgroup, the GG/GA + AA value was 0.317 for the EH group and 0.314 for the control. The pooled OR for the GG/GA + AA value was 1.01 (95% CI: 0.82–1.25, *P*_heterogeneity_ = 0.11, *I*^2^ = 50.7%). The association between the *ACE2* G8790A gene polymorphism and EH in the women was not significant (*P* = 0.92; Figure 3; Table 1).

**Figure 4:**
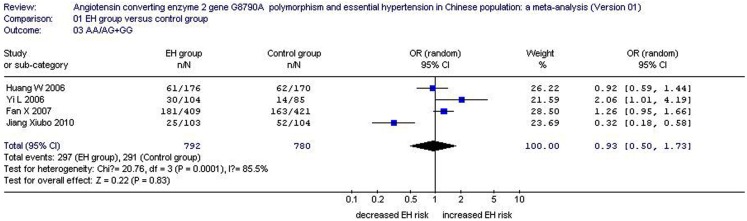
**Forest plot of the recessive genetic model of essential hypertension associated with *ACE2* G8790A gene polymorphism in female population (AA/GA + GG value of angiotensin converting enzyme 2 gene)**.

Under a recessive model of inheritance in the female subgroup, the AA/AG + GG value was 0.375 for the EH group and 0.373 for the control. The pooled OR for the AA/AG + GG value was 0.93 (95% CI: 0.50–1.73, *P*_heterogeneity_ = 0.0001, *I*^2^ = 85.5%). There was no significant association between the *ACE2* G8790A gene polymorphism and EH in the women (*P* = 0.83; Figure 4; Table 1).

Meta-regression was conducted to explore the potential sources of heterogeneity in the male subgroup under the allelic genetic model of inheritance. The confounding factors included the publication year, study region, ethnicity, G genotype number of EH group sample size (G1), and G genotype number of control group sample size (G0). All these confounding factors could explain the heterogeneity (*P* < 0.05), but publication year was the most important (*P* = 0.017; Table 2).

**Table 2:** **The meta-regression results among eight studies in the male subgroup under the allelic genetic model**.

Item	Coefficient	Standard error	*T* value	*P* value	95% Confidence interval
Weight	−0.1845581	0.014793	−12.48	0.051	−0.3725206 to 0.0034044
Publication year	−0.5106332	0.0132544	−38.53	0.017*	−0.6790462 to −0.3422202
Region	−0.8178791	0.0265906	−30.76	0.021*	−1.155745 to −0.480013
G genotype number of EH group size	0.0111473	0.0005284	21.10	0.030*	0.0044336 to 0.017861
Ethnicity	−0.3915033	0.0197832	−19.79	0.032*	−0.6428729 to −0.1401337
G genotype number of control group size	−0.0156601	0.0007113	−22.02	0.029*	−0.0246984 to −0.0066219
Cons	1028.613	26.69087	38.54	0.017	689.4735 to 1367.753

*P < 0.05.

*Coefficient: regression coefficient*.

*The regression coefficients are the estimated increase in the lnOR per unit increase in the covariates. Cons, constant item*.

**Figure 5:**
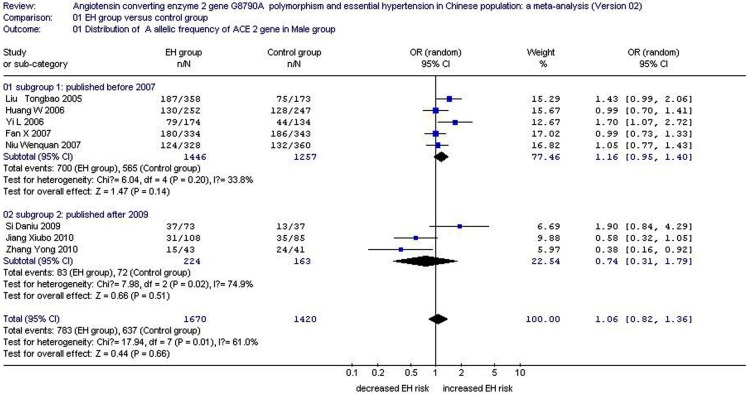
**Forest plot of EH associated with *ACE2* G8790A gene polymorphism stratified by publication year in male subgroup (distribution of A allelic frequency of *ACE2* gene)**.

In the subsection analysis stratified by the publication year, Subsection 1 was defined as publication year ranging from 2005 to 2007. Studies with publication year ranging from 2009 to 2010 were defined as Subsection 2. In Subsection one of five studies, the pooled OR was 1.16 (95% CI: 0.95–1.40, *P*_heterogeneity_ = 0.20, *I*^2^ = 33.8%). In Subsection 2 of three studies, the pooled OR was 0.74 (95% CI: 0.31–1.79, *P*_heterogeneity_ = 0.02, *I*^2^ = 74.9%). The subsection analysis indicated that the heterogeneity in Subsection 2 was higher than that in Subsection 1. Therefore, studies on Subsection 2 need further improvement (Table 3; Figure 5).

**Table 3:** **Subsection analysis summary of the allelic genetic model stratified by publication year in the male subgroup**.

Subsection	Studies number	Weight (%)	Pooled OR (95% CI)	*Z* (*P*)	*I*^2^ (%)
Subsection 1	5	77.46	1.16 (0.95–1.40)	1.47 (*P* = 0.14)	33.8
Subsection 2	3	22.54	0.74 (0.31–1.79)	0.66 (*P* = 0.51)	74.9
Whole population	8	100.0	1.06 (0.82–1.36)	0.44 (*P* = 0.66)	61.0

*Subsection 1: published before 2007*.

*Subsection 2: published after 2009*.

### Bias diagnostics

**Figure 6:**
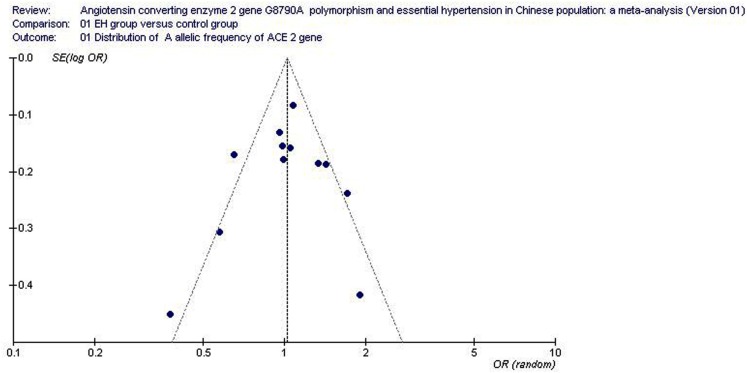
**Funnel plot for studies of the association of essential hypertension and ACE2 G8790A gene polymorphism (distribution of A allelic frequency of *ACE2* gene)**. The horizontal and vertical axis correspond to the OR and confidence limits. OR, odds ratio; SE, standard error.

No visual evidence of publication bias was found from the funnel plot (Figure 6). There was also no significant difference in the Egger’s test under the allelic genetic model, which implied that the publication bias was low in the current meta-analysis (*P* = 0.658, *T* = 0.46).

## Discussion

Essential hypertension is a complex disease caused by a combination of genetic and environmental factors. Crackower et al. (2002) have found that although the gene knockout of *ACE2* does not affect blood pressure regulation, the serum Ang II level significantly increases. The cardiovascular and renal effects of Ang II are the most important long-term blood pressure regulation mechanisms. They have also found that *ACE2* knockout mice exhibit the cardiac abnormalities similar with cardiac stunning in humans.

The G8790A gene polymorphism of *ACE2* is the restriction enzyme cutting site located on the fourth base of the third intron. The code is ranked as rs2285666 in the United States Biotechnology Information Center. mRNA and protein expression levels change in cardiovascular diseases and diabetes mellitus (DM; Ribeiro-Oliveira et al., 2008). Hence, the G8790A gene polymorphism may participate in the onset of such illnesses as hypertension, cardiac dysfunction, and DM. The G8790A gene polymorphism is situated in the intron adjoined to the extron, suggesting that this locus could alter mRNA alternate splicing and affect *ACE2* gene expression. The latter led to changes in the serum content of Ang 1–7 that inhibited vasoconstriction and cell proliferation (Kramkowski et al., 2006).

Given the location of the *ACE2* gene in X chromosomes, the association of the G8790A gene polymorphism and EH was classified and analyzed according to gender. In the present meta-analysis, under the premise that age and gender factors were controlled, no significant association was found between the *ACE2* G8790A gene polymorphism and EH under allelic, dominant, and recessive genetic models. There was no significant difference between the EH and control groups in either male or female subgroups. The current meta-analysis suggested that the A allele of the *ACE2* G8790A gene polymorphism might not be related to increased risk for EH.

In the meta-regression of the confounding factor, the publication year was considered as the most important heterogeneity source. The publication year before 2007 indicated lower heterogeneity, and suggested that the early studies have the smaller heterogeneity than the latter studies.

The results of the current research conformed to those of Zhou and Yang (2009) who meta-analyzed the *ACE2* G8790A gene polymorphism and EH in a Chinese Han population. The *ACE2* G8790A gene polymorphism might not be a genetic risk factor for EH in the said population. However, not all of the included studies have followed the HWE. For instance, the study of Liu et al. (2005) has deviated from the HWE. Hence, this study was excluded in the current meta-analysis. In addition, three genetic models, namely, allelic, dominant, and recessive genetic models, were adopted in the present study to analyze the research results. In contrast, only the allelic genetic model was used in Zhou’s work. Regarding Lu’s et al. (2012) work, the limitation also existed. The studies against HWE were also included in the female subgroup in their meta-analysis (Liu et al., 2005; Niu et al., 2007; Si et al., 2009; Zhang et al., 2010). Moreover, another included study was not associated with EH but orthostatic hypertension (Fan et al., 2009). Given these limitations, Lu’s conclusion seemed not credible.

In conclusion, the current meta-analysis suggested that the *ACE2* G8790A gene polymorphism was not associated with susceptibility to EH in the Chinese population. Further studies should use higher investigation sample sizes. Given the relatively small sample size used in the present study, further research should be performed to test the abovementioned conclusion.

## Conflict of Interest Statement

The author declares that the research was conducted in the absence of any commercial or financial relationships that could be construed as a potential conflict of interest.

## Supplementary Material

The Supplementary Material for this article can be found online at http://www.frontiersin.org/Vascular_Physiology/10.3389/fphys.2012.00364/abstract

Supplementary Table S1 **Characteristics of the investigated studies of the association between the ACE2 G8790A gene polymorphism and EH of the male subgroup**.

Supplementary Table S2 **RT-PCR primers utilized for each gene in the RT-PCR analysis**.

## References

[B1] Benjafield A. V. Wang W. Y. Morris B. J. (2004). No association of angiotensin-converting enzyme 2 gene (ACE2) polymorphisms with essential hypertension. Am. J. Hypertens. 17, 624–628 10.1016/j.amjhyper.2004.02.022 15233982 PMC7110370

[B2] Cochran W. G. (1968). The effectiveness of adjustment by subclassification in removing bias in observational studies. Biometrics 24, 295–313 10.2307/2528036 5683871

[B3] Crackower M. A. Sarao R. Oudit G. Y. Yagil C. Kozieradzki I. Scanga S. E. Oliveira-dos-Santos A. J. da Costa J. Zhang L. Pei Y. Scholey J. Ferrario C. M. Manoukian A. S. Chappell M. C. Backx P. H. Yagil Y. Penninger J. M. (2002). Angiotensin-converting enzyme 2 is an essential regulator of heart function. Nature 417, 822–828 10.1038/nature00786 12075344

[B4] DerSimonian R. Laird N. (1986). Meta-analysis in clinical trials. Control. Clin. Trials 7, 177–188 10.1016/0197-2456(86)90046-2 3802833

[B5] Donoghue M. Hsieh F. Baronas E. Godbout K. Gosselin M. Stagliano N. Donovan M. Woolf B. Robison K. Jeyaseelan R. Breitbart R. E. Acton S. (2000). A novel angiotensin-converting enzyme-related carboxypeptidase (ACE2) converts angiotensin I to angiotensin 1–9. Circ. Res. 87, E1–E9 10.1161/01.RES.87.1.1 10969042

[B6] Egger M. Davey Smith G. Schneider M. Minder C. (1997). Bias in meta-analysis detected by a simple, graphical test. Br. Med. J. 315, 629–634 10.1136/bmj.315.7119.1371 9310563 PMC2127453

[B7] Fan X. Wang Y. Sun K. Zhang W. Yang X. Wang S. Zhen Y. Wang J. Li W. Han Y. Liu T. Wang X. Chen J. Wu H. Hui R. T. (2007). Polymorphisms of ACE2 gene are associated with essential hypertension and antihypertensive effects of Captopril in women. Clin. Pharmacol. Ther. 82, 1–10 10.1038/sj.clpt.6100214 17473847

[B8] Fan X. H. Wang Y. B. Wang H. Sun K. Zhang W. L. Song X. D. Cheng J. Z. Wu H. Y. Zhou X. L. Hui R. T. (2009). Polymorphisms of angiotensin-converting enzyme (ACE) and ACE2 are not associated with orthostatic blood pressure dysregulation in hypertensive patients. Acta Pharmacol. Sin. 30, 1237–1244 10.1038/aps.2009.110 19684612 PMC4007186

[B9] Hang W. Yang W. Wang Y. Zhao Q. Gu D. Chen R. (2006). Association study of angiotensin-converting enzyme 2 gene (ACE2) polymorphisms and essential hypertension in northern Han Chinese. J. Hum. Hypertens. 20, 968–971 10.1038/sj.jhh.1002090 17024138

[B10] Jiang X. B. Zhang D. F. Jiang W. J. Wang S. J. Song X. Li D. J. Jiang Z. C. (2010). Association of angiotensin-converting enzyme 2 gene G8790A polymorphism with essential hypertension in Han Chinese. Chin. Gen. Prac. 13, 3274–3277

[B11] Kramkowski K. Mogielnicki A. Buczko W. (2006). The physiological significance of the alternative pathways of angiotensin II production. J. Physiol. Pharmacol. 57, 529–539 17229979

[B12] Liu T. B. Shang H. P. Zhang K. X. Chen L. H. Zhu X. L. Zhang Y. Zhu D. L. Huang W. (2005). Association of angiotensin I converting enzyme 2 gene polymorphism with essential hypertension in Chinese. Chin. J. Med. Genet. 22, 569–571 16215952

[B13] Lu N. Yang Y. Wang Y. Liu Y. Fu G. Chen D. Dai H. Fan X. Hui R. Zheng Y. (2012). ACE2 gene polymorphism and essential hypertension: an updated meta-analysis involving 11,051 subjects. Mol. Biol. Rep. 39, 6581–6589 10.1007/s11033-012-1483-5 22297693

[B14] Mantel N. Haenszel W. (1959). Statistical aspects of the analysis of data from retrospective studies of disease. J. Natl. Cancer Inst. 22, 719–748 13655060

[B15] Niu W. Q. Qi Y. Hou S. Q. Zhou W. Y. Qiu C. C. (2007). Correlation of angiotensin-converting enzyme 2 gene polymorphisms with stage 2 hypertension in Han Chinese. Transl. Res. 150, 374–380 10.1016/j.trsl.2007.06.002 18022600

[B16] Ribeiro-Oliveira A. Jr. Nogueira A. I. Pereira R. M. Boas W. W. Dos Santos R. A. Simões e Silva A. C. (2008). The renin-angiotensin system and diabetes: an update. Vasc. Health Risk Manag. 4, 787–803 19065996 PMC2597759

[B17] Si D. N. Li L. Chui T. X. (2009). Association of angiotensin I converting enzyme 2 gene polymorphism and essential hypertension with diabetes mellitus. Chin. J. Pract. Nerv. Dis. 12, 38–40

[B18] Tipnis S. R. Hooper N. M. Hyde R. Karran E. Christie G. Turner A. J. (2000). A human homolog of angiotensin-converting enzyme. Cloning and functional expression as a captopril-insensitive carboxypeptidase. J. Biol. Chem. 275, 33238–33243 10.1074/jbc.M002615200 10924499

[B19] Yi L. Gu Y. H. Wang X. L. An L. Z. Xie X. D. Shao W. Ma Y. Fang J. R. An Y. D. Zhang D. L. (2006). Association of ACE, ACE2 and UTS2 polymorphisms with essential hypertension in Han and Dongxiang populations from north-western China. J. Int. Med. Res. 34, 272–283 16866021 10.1177/147323000603400306

[B20] Zhang H. M. Fan X. H. Han Y. F. Li W. J. Yang X. M. Wang S. X. Zhou X. L. Wu H. Y. Hui R. T. (2008). Polymorphism interaction between angiotensin-conversion enzyme 2 gene and angiotensin-conversion enzyme gene on the risk of essential hypertension in women. Chin. Circ. J. 23, 355–359

[B21] Zhang Y. Wu K. Jin S. J. Zhou D. F. Zhang Y. X. Chen S. H. Li L. J. Wang Z. Xi J. L. Chen Z. Q. (2010). Association of angiotensin I converting enzyme 2 gene polymorphism and essential hypertension in Hainan Li Chinese population. Chin. J. Gerontol. 30, 1029–1230

[B22] Zhong J. Yan Z. Liu D. Ni Y. Zhao Z. Zhu S. Tepel M. Zhu Z. (2006). Association of angiotensin-converting enzyme 2 gene A/G polymorphism and elevated blood pressure in Chinese patients with metabolic syndrome. J. Lab. Clin. Med. 147, 91–95 10.1016/j.lab.2005.10.001 16459167 PMC7127450

[B23] Zhou J. B. Yang J. K. (2009). Meta-analysis of association of ACE2 G8790A polymorphism with Chinese Han essential hypertension. J. Renin Angiotensin Aldosterone Syst. 10, 31–34 10.1177/1470320309103047 19286756

