# Supplementary material for: Lack of Association of ACE2 G8790A Gene Mutation with Essential Hypertension in the Chinese Population: A Meta-Analysis Involving 5260 Subjects
Source: Front Physiol. 2012 Sep 12;3:364. doi: 10.3389/fphys.2012.00364 (PMC3439858; doi:10.3389/fphys.2012.00364)
Supplement: Supplementary Table S1 — Characteristics of the investigated studies of the association between the ACE2 G8790A gene polymorphism and EH of the male subgroup. [file 31838_Li_Presentation1.PDF]

Supplement 1. Characteristics of the investigated studies of the association between the ACE2 G8790A gene polymorphism and EH of the male subgroup

Author	Year	Region	Ethnicity	Control		EH		Matching criteria	sample size (EH/control)
				G	A	G	A		
Liu TB [14]	2005	Shandong	Han	98	75	171	187	Age, BMI, ethnicity	358/173
Huang W [17]	2006	Hunan	Han	119	128	122	130	Age,,ethnicity,area	252/247
Yi L [18]	2006	Gansu	Han, Dongxiang	90	44	95	79	Age,sex,BMI	174/134
Fan X[19]	2007	Beijing	Han	157	186	154	180	Age,,ethnicity	334/343
Niu WQ[15]	2007	Beijing	Han	228	132	204	124	Age, ethnicity	328/360
Si DN[6]	2009	Henan	Han	24	13	36	37	Age, BMI,ethnicity	73/37
Jiang XB[5]	2010	Shandong	Han	50	35	77	31	Ethnicity	108/85
Zhang Y[16]	2010	Hainan	Li	17	24	28	15	Age, BMI,ethnicity	43/41

Abbreviations:

BMI: body mass index;

The polymerase chain reaction-restriction fragment length polymorphism (PCR-RFLP) geno-typing method and case-control study design were adopted in all of the above studies

Supplement 2. Characteristics of the investigated studies of the association between the *ACE2* G8790A gene polymorphism and EH of the female subgroup

Author	Year	Region	Ethnicity	Control			EH			Matching criteria	sample size
				GG	GA	AA	GG	GA	AA		(EH/control)
Huang W [17]	2006	Hunan	Han	55	115	62	49	127	61	Age,ethnicity,area	237/232
Yi L [18]	2006	Gansu	Han, Dongxiang	29	56	14	42	62	30	Age,sex,BMI	144/99
Fan X[19]	2007	Beijing	Han	135	286	163	131	278	181	Age,ethnicity	590/584
Jiang XB[5]	2010	Shandong	Han	37	67	52	40	63	25	Ethnicity	128/156

Abbreviations:

BMI: body mass index;

The polymerase chain reaction-restriction fragment length polymorphism (PCR-RFLP) geno-typing method and case-control study design were adopted in all of the above studies
